# Post-conization pathological upgrading and outcomes of 466 patients with low-grade cervical intraepithelial neoplasia

**DOI:** 10.3389/fonc.2024.1449080

**Published:** 2024-09-11

**Authors:** Yulin Guo, Hongning Cai, Qiuzi Peng, Ying Wang, Lu Li, Miao Zou, Jinyue Guo, Chaonan Wang, Xufeng Wu, Quanfu Ma

**Affiliations:** ^1^ Cervical Cancer Control Center of Hubei Province, Maternal and Child Health Hospital of Hubei Province, Wuhan, China; ^2^ Hubei Clinical Medical Research Center for Gynecologic Malignancy, Maternal and Child Health Hospital of Hubei Province, Wuhan, China

**Keywords:** cervical intraepithelial neoplasia, conization, cytology, follow-up, human papillomavirus, pathological upgrading

## Abstract

**Introduction:**

The management of patients with low-grade cervical intraepithelial neoplasia (CIN1) remains controversial. We analyzed the pathological upgrading rates of patients with CIN1 undergoing conization, identifying influencing factors, and compared their outcomes to those of patients with CIN1 receiving follow-up only.

**Methods:**

This retrospective study included 466 patients with CIN1 confirmed by histopathology and treated with conization. Postoperative pathological upgrading was determined and its influencing factors were identified. We also analyzed post-conization outcomes, examining the rate of persistent/recurrent CIN1 and its influencing factors, and comparing these results to those of patients receiving follow-up only.

**Results:**

The pathological upgrading rate of patients with CIN1 after conization was 21.03% (98/466), and the influencing factors were preoperative high-risk human papillomavirus (HR-HPV) infection and cytological results. The upgrading rates of HR-HPV positive and negative patients were 22.05% and 0.00%, respectively (*χ* ^2^ = 5.03, *P*=0.03). The upgrading rate of patients with cytological results negative for intraepithelial lesion malignancy was 10.94%, while the upgrading rates of atypical squamous cells, cannot exclude high-grade lesion(ASC-H) and high-grade squamous intraepithelial lesion(HSIL) groups were 47.37% and 52.94%, respectively (*χ* ^2 =^ 22.7, *P*=0.03). Persistent/recurrent CIN1 rates in the conization group were 21.24%, 15.97%, and 6.67% at 6, 12, and 24 months, respectively, significantly lower than those in the follow-up only group. The CIN2 progression rate in the conization group (0.26%) during the 24-month follow-up period was also significantly lower than that in the follow-up only group (15.15%; *χ* ^2 =^ 51.68, *P*<0.01). The only factor influencing postoperative persistent/recurrent CIN1 was preoperative HR-HPV status. No patients who were HR-HPV negative preoperatively exhibited persistent/recurrent CIN1, compared with 25.55% of those who were HR-HPV positive preoperatively (*χ* ^2^ = 4.40, *P*=0.04).

**Discussion:**

The risk of progression to CIN2+ in the medium term is higher in patients with CIN1 receiving follow-up than in those undergoing conization. Doctors should refer to the guidelines but comprehensively consider age, fertility requirements, preoperative HR-HPV and cytological results, follow-up conditions, and other factors to select the most appropriate treatment strategy for patients with CIN1.

## 1 Introduction

Widespread cervical cancer screening in China has increased the detection of cervical intraepithelial neoplasia (CIN), including low-grade CIN (CIN1). Although CIN1 can develop into cervical cancer, its natural regression rate is high, and the risk of progression to invasive cancer is low; the optimal treatment strategy for CIN1 is therefore unclear (1). In 2001, the American Society for Colposcopy and Cervical Pathology (ASCCP) recommended that the treatment strategy should be determined by vaginal examination, with surgical treatment performed when vaginal examination results are unsatisfactory. If colposcopy is satisfactory, patients should be followed up, although surgery remains an option (2). The 2006 guidelines were updated to recommend regular follow-up for patients with CIN1; for those with high-grade squamous intraepithelial lesions (HSIL) or atypical glandular cells not otherwise specified, as determined by cytology, diagnostic conization or follow-up strategies can be adopted (3). The 2012 update was largely consistent with the 2006 guidelines, recommending observation rather than treatment, although the follow-up recommendations were revised to include human papillomavirus (HPV) testing combined with cytology, and the screening interval was extended to 12 months. In addition, a minimum threshold of two years was proposed before beginning CIN1 treatment (4). The 2019 update again advocated observation rather than treatment (5). The changes in the guidelines over the past 20 years reflect an increased understanding of the biological behavior of CIN1 and improved monitoring methods, allowing more conservative management of CIN1.

ASCCP recommendations regarding CIN1 are applicable in developed countries, which benefit from advanced cytological screening programs, cell pathology experts, reliable colposcopy, and pathological diagnostic technologies, all of which are less widely available in developing countries. Therefore, allocating patients with CIN1 in developing countries for follow-up only may increase the CIN2+ missed diagnosis rate; the risk of progression and compliance issues must also be considered.

Therefore, we question whether it is safe to manage patients with CIN1 in developing countries or regions according to the 2019 ASCCP guidelines. To answer this question, we conducted a retrospective study of patients with CIN1 admitted to our center over a 14-year period. We analyzed pathological upgrading in patients treated with conization, explored the influencing factors, and compared the outcomes of these patients with those of patients who underwent follow-up only.

## 2 Materials and methods

### 2.1 Patients

The medical records of patients admitted to Hubei Cervical Cancer Prevention and Treatment Center between January 1, 2008, and December 31, 2021, who had a diagnosis of CIN1 histologically confirmed and were treated with conization were retrospectively reviewed. The inclusion criteria were as follows: (1) initial screening with cytology and/or high-risk (HR)-HPV testing, (2) diagnosis of CIN1 confirmed by pathology on a colposcopy-directed biopsy, (3) treatment with conization surgery, (4) not pregnant, and (5) no previous history of cervical disease ablation or surgical resection. To further explore the necessity of conization surgery for CIN1, we also reviewed the records of 66 patients with CIN1 who received regular informed follow-ups at our center from 2016 to 2022. This study was approved by the Ethics Review Committee of Hubei Maternal and Child Health Hospital, and all patients provided written informed consent.

### 2.2 HPV and cytological testing

HPV testing in our center was performed using the Cervista™ HPV HR test (Hologic, Inc., Marlborough, MA, USA), an *in vitro* diagnostic test for the detection of DNA from 14 types of HR-HPV (16, 18, 31, 33, 35, 39, 45, 51, 52, 56, 58, 59, 66, and 68) with results were divided into A9, A7, and A5/6 groups, and the Digene Hybrid Capture 2 test (Qiagen, Hilden, Germany), which detects 13 oncogenic genotypes (16, 18, 31, 33, 35, 39, 45, 51, 52, 56, 58, 59, and 68), with results classified as positive at a relative light unit value ≥1 pg/mL, and the Cobas 4800 test (Roche Molecular Systems, Pleasanton, CA, USA) which is able to detect the HPV16 and HPV18 genotypes separately, as well as detecting a group of hrHPV genotypes (HPV31, 33, 35, 39, 45, 51, 52, 56, 58, 59, 66, and 68). The Kaipu HPV 21 typing test (Guangzhou Kaipu Biotechnology Co., Ltd., Guangzhou, China), which classified HPV into 15 high-risk types (16, 18, 31, 33, 35, 39, 45, 51, 52, 53, 56, 58, 59, 66, and 68) and 6 low-risk types (6, 11, 42, 43, 44, and cp8304), was typically used for referred cases.

Cytology testing comprised liquid-based cytology testing using the ThinPrep^®^ 2000 system (Hologic, Inc.). Final cytological diagnosis was made using the Bethesda system (6, 7). Positive cytology findings included atypical squamous cells of unknown significance (ASC-US), ASC-H, low-grade squamous intraepithelial lesion (LSIL), and HSIL.

### 2.3 Colposcopy-directed biopsy and histopathological examination

A solution containing normal saline, 5% acetic acid, and 5% iodine was administered to the cervix to identify the transformation zone (TZ) type and any abnormal features, and to perform colposcopy-directed biopsy. In the case of type III TZ, multipoint biopsy and/or endocervical curettage was performed, taking into consideration the medical history, cytology test results, and HR-HPV test results. Each biopsy specimen was stored separately in 10% neutral formalin prior to pathological examination, with diagnosis confirmed following simultaneous analysis by two specialist pathologists. In cases of disagreement, the director of the pathology department was consulted.

Some patients with CIN1 were referred from other hospitals. For these patients, basic information on primary screening, colposcopy, and biopsy pathology was obtained from outpatient medical records. The findings were confirmed by two pathologists prior to patient admission.

### 2.4 Conization surgery

Conization surgery was mainly performed using the loop electrosurgical excision procedure (LEEP), with a small proportion of patients undergoing cold knife conization. Preoperative re-evaluation was conducted to obtain vaginal endoscopic images, and the range of conization was determined based on the type and extent of the TZ. The resection length of type I and II TZs was approximately 10–15 mm, and the resection length of type III TZs was 15–18 mm. Since 2018, preoperative evaluation has included ultrasound examinations to measure the length of the cervical canal. After careful consideration, the surgeon selected the surgical method and scope. Specimens were collected from 12 or 24 sites, embedded in paraffin, sliced into 4 μm sections, stained with hematoxylin and eosin, and examined under a light microscope. Suspected cases of CIN2+ were selected for immunohistochemistry, mainly staining for P16/Ki67. Pathological diagnosis was performed following simultaneous analysis by two senior pathologists.

### 2.5 Follow-up

After conization, patients with CIN1 or negative pathology were transferred to the outpatient clinic for regular follow-up. Patients with an upgraded pathological diagnosis after conization received further treatment in accordance with the relevant guidelines and degree of upgrade (8, 9). Post-conization follow-up was performed at 3, 6, 9, 12, 18, and 24 months, and annually thereafter. Cervical cytology and HR-HPV tests were performed at each visit, and patients with abnormal cytology findings and those who were HR-HPV-positive were referred for colposcopy-guided biopsy. Some patients who were re-examined at local hospitals were followed-up by telephone. All follow-up results were recorded.

Persistent/recurrent CIN2/3 was diagnosed based on a histological diagnosis or the results of at least two cytology tests. The presence of a histological diagnosis of CIN1, a cytology result indicating ASC or LSIL, a single cytology finding of HSIL without histological confirmation, or a positive HR-HPV test result with negative cytology and/or histology results during follow-up were considered potential indications of persistent/recurrent CIN1 (10). Patients with a negative cytology test result, a negative HR-HPV test result, and, if performed, negative histology findings were considered to have no persistent/recurrent disease. After two years of consecutive negative results, follow-up frequency was reduced to once a year.

### 2.6 Statistical analysis

Data were analyzed and processed using SPSS software version 24.0 (IBM Corp., Armonk, NY, USA). Count data are expressed as frequencies and intergroup comparisons were performed using chi-squared tests. *P*<0.05 was considered to indicate a statistically significant difference.

## 3 Results

### 3.1 Patient characteristics

A total of 466 eligible patients were included in the study, with an average age of 41.11 ± 10.70 years. The majority of patients (70.60%) were diagnosed following routine physical examinations. HPV testing was performed on 408 patients, with 390 (95.59%) testing positive. Cytological testing was conducted on 384 patients, with 320 (83.33%) showing with abnormal results. Regarding the conization method, 433 patients (92.92%) underwent LEEP, while 33 (7.08%) patients cold knife conization (**Table 1**).

**Table 1:** Characteristics of 466 patients with low-grade cervical intraepithelial neoplasia.

Characteristic	N	Percentage
Age (years)		
<30	67	14.38%
30–39	147	31.54%
40–49	152	32.62%
≥50	100	21.46%
Reason for presentation		
Routine medical checkup	329	70.60%
Contact bleeding	67	14.38%
Abnormal vaginal discharge	54	11.59%
Other	16	3.43%
High risk-human papillomavirus test result (N=408)		
Negative	18	4.41%
Positive	390	95.59%
Cytology findings (N=384)		
Negative for intraepithelial lesion malignancy	64	16.67%
Atypical squamous cells of unknown significance	169	44.01%
Low-grade squamous intraepithelial lesion	115	29.95%
Atypical squamous cells, cannot exclude high-grade lesion	19	4.95%
High-grade squamous intraepithelial lesion	17	4.43%
Conization method		
Loop electrosurgical excision procedure	433	92.92%
Cold knife conization	33	7.08%

### 3.2 Pathological upgrading after conization

After conization, 144 and 224 patients received a pathological diagnosis of CIN1 and chronic cervicitis, respectively, and the remaining 98 patients received CIN2+ pathological upgrading. Of these 98 cases, 94 were of CIN2/3, 2 were of adenocarcinoma *in situ*, and 2 were of squamous cell carcinoma (pathological upgrading rate, 21.03%).

### 3.3 Factors affecting pathological upgrading after conization

Preoperative HR-HPV and cytological test results were identified as influencing factors of pathological upgrading following conization. The upgrading rate of patients who tested positive for HR-HPV was 22.05%, while none of the 18 patients who tested negative were upgraded; this difference was statistically significant (*χ* ^2^ = 5.03, *P*=0.03). Among the 64 patients with preoperative cytology results NILM, only 7 were upgraded (upgrading rate, 10.94%). In contrast, pathological upgrading was performed in 23.67%, 18.26%, 47.37%, and 52.94% of patients with cytology findings of ASC-US, LSIL, ASC-H, and HSIL, respectively, with the upgrading rate increasing with lesion severity. Other analyzed factors, such as age, reason for presentation, pregnancy and childbirth history, and cervical erosion were not related to postoperative pathological upgrading (**Table 2**).

**Table 2:** Analysis of factors influencing postoperative pathological upgrading.

Factor	Postoperative pathological upgrading		χ^2^	*P*
	No (N=368)	Yes (N=98)		
Age (years)				
<30	56 (83.58%)	11 (16.42%)	3.35	0.34
30–39	119 (80.95%)	28 (19.05%)		
40–49	120 (78.95%)	32 (21.05%)		
≥50	73 (73.00%)	27 (27.00%)		
Reason for presentation				
Routine medical checkup	256 (77.81%)	73 (22.19%)	1.37	0.71
Contact bleeding	55 (82.09%)	12 (17.91%)		
Abnormal vaginal discharge	43 (79.63%)	11 (20.37%)		
Other	14 (87.50%)	2 (12.50%)		
HR-HPV test result (N=408)				
Negative	18 (100.00%)	0 (0.00%)	5.03	0.03
Positive	304 (77.95%)	86 (22.05%)		
HR-HPV type (N=224)				
16/18+	58 (73.42%)	21 (26.58%)	3.21	0.07
12+	121 (83.45%)	24 (16.55%)		
HR-HPV load (N=82)				
<100	17 (80.95%)	4 (19.05%)	4.01	0.13
100–500	12 (70.59%)	5 (29.41%)		
>500	40 (90.91%)	4 (9.09%)		
Cytology findings (384)				
NILM	57 (89.06%)	7 (10.94%)	22.07	<0.01
ASC-US	129 (76.33%)	40 (23.67%)		
LSIL	94 (81.74%)	21 (18.26%)		
ASC-H	10 (52.63%)	9 (47.37%)		
HSIL	8 (47.06%)	9 (52.94%)		
Cervical erosion				
None	204 (80.31%)	50 (19.69%)	4.57	0.21
Mild	112 (78.32%)	31 (21.68%)		
Moderate	42 (80.77%)	10 (19.23%)		
Severe	10 (58.82%)	7 (41.18%)		
Gravidity				
0	31 (83.78%)	6 (16.22%)	4.00	0.13
1–2	142 (83.04%)	29 (16.96%)		
≥3	195 (75.58%)	63 (24.42%)		
Parity				
0	55 (80.88%)	13 (19.12%)	0.39	0.82
1–2	286 (78.36%)	79 (21.64%)		
≥3	27 (81.82%)	6 (18.18%)		

ASC-H, atypical squamous cells, cannot exclude high-grade lesion; ASC-US, atypical squamous cells of unknown signiﬁcance; HR-HPV, high risk-human papillomavirus; HSIL, high-grade squamous intraepithelial lesion; LSIL, low-grade squamous intraepithelial lesion; NILM; negative for intraepithelial lesion malignancy.

A total of 353 patients underwent both preoperative HPV testing and cytological screening; these patients were further stratified according to the results. Analysis was performed, with the HPV (+)/NILM group serving as a reference. Postoperative pathological upgrades rates were significantly higher in the HPV (+)/ASC-H (50%; *P*<0.01) and HPV (+)/HSIL (60%; *P*<0.01) groups than in the HPV (+)/NILM group (11.86%) (**Table 3**).

**Table 3:** Pathological upgrading of patients with low-grade cervical intraepithelial neoplasia after conization according to initial screening.

Screening group	Pathological upgrading after conization		*χ* ^2^	*P*
	No	Yes		
HPV(+)/NILM	52 (88.14%)	7 (11.86%)	–	–
HPV(+)/ASC-US	112 (78.87%)	30 (21.13%)	2.38	0.12
HPV(+)/LSIL	87 (82.08%)	19 (17.92%)	1.05	0.31
HPV(+)/ASC-H	9 (50.00%)	9 (50.00%)	12.19	<0.01
HPV(+)/HSIL	6 (50.00%)	9 (60.00%)	16.35	<0.01
HPV(-)	13 (100.00%)	0 (0.00%)	1.71	0.34

ASC-H, atypical squamous cells, cannot exclude high-grade lesion; ASC-US, atypical squamous cells of unknown signiﬁcance; HPV, human papillomavirus; HSIL, high-grade squamous intraepithelial lesion; LSIL, low-grade squamous intraepithelial lesion; NILM; negative for intraepithelial lesion malignancy.

### 3.4 Persistent/recurrent CIN after conization

A total of 379 patients were followed-up after conization. According to the classification described by Rodriguez-Manfredi et al. (10), 84 of these had persistent/recurrent CIN1 and 1 had persistent/recurrent CIN2. The majority of patients with persistent/recurrent CIN1 were positive for HR-HPV. The rates of persistent/recurrent CIN1 at 3, 6, 12, and 24 months postoperatively were 22.16% (84/379), 21.24% (65/306), 15.97% (42/263), and 6.67% (14/210), respectively.

We further analyzed the factors influencing the three-month persistent/recurrent CIN1 rates after conization, identifying only preoperative HR-HPV status as a possible influencing factor. Of the 13 patients negative for HPV preoperatively, none developed persistent/recurrent CIN1 within three months of conization, compared to 82 out of 321 patients positive for HPV preoperatively (25.55%) (*χ* ^2^ = 4.40, *P*=0.04) (**Table 4**).

**Table 4:** Analysis of factors influencing the development of persistent/recurrent CIN1 within three months of conization.

Factor	Persistent/recurrent CIN1		*χ* ^2^	*P*
	No	Yes		
Age (years)				
<30	43 (74.14%)	15 (25.86%)	0.98	0.81
30–39	100 (77.52%)	29 (22.48%)		
40–49	95 (80.51%)	23 (19.49%)		
≥50	57 (77.03%)	17 (22.97%)		
Reason for presentation				
Routine medical check up	208 (76.19%)	65 (23.81%)	3.00	0.39
Contact bleeding	38 (77.55%)	11 (22.45%)		
Abnormal vaginal discharge	37 (84.09%)	7 (15.91%)		
Other	12 (92.31%)	1 (7.69%)		
HR-HPV test result (N=334)				
Negative	13 (100.00%)	0 (0.00%)	4.40	0.04
Positive	239 (74.45%)	82 (25.55%)		
Cytology findings (N=320)				
NILM	42 (79.25%)	11 (20.75%)	3.43	0.49
ASC-US	111 (79.29%)	29 (20.71%)		
LSIL	66 (70.21%)	28 (29.79%)		
ASC-H	14 (82.35%)	3 (17.65%)		
HSIL	13 (81.25%)	3 (18.75%)		
Conization method				
LEEP	271 (76.99%)	81 (23.01%)	2.06	0.15
CKC	24 (88.89%)	3 (11.11%)		
Conization results				
Negative	140 (80.00%)	35 (20.00%)	1.32	0.52
CIN1	90 (74.38%)	31 (25.62%)		
CIN2+	65 (78.31%)	18 (21.69%)		
Margins				
Negative	280 (77.13%)	83 (22.87%)	2.45	0.12
Positive	15 (93.75%)	1 (6.25%)		

ASC-H, atypical squamous cells, cannot exclude high-grade lesion; ASC-US, atypical squamous cells of unknown signiﬁcance; CIN, cervical intraepithelial neoplasia; CKC, cold knife conization; HR-HPV, high risk-human papillomavirus; HSIL, high-grade squamous intraepithelial lesion; LEEP, loop electrosurgical excision procedure; LSIL, low-grade squamous intraepithelial lesion; NILM; negative for intraepithelial lesion malignancy.

### 3.5 Outcomes patients receiving surgery or follow-up

To further explore the necessity of conization surgery for CIN1, we compared the outcomes of patients who underwent conization with those of patients who instead received informed follow-up at our center from 2016 to 2022. There were 379 and 66 patients in the conization and follow-up groups, respectively. The incidence of persistent/recurrent CIN1 in the conization group after surgery was significantly lower than that in the follow-up group at 6, 12, and 24 months (**Table 5**). Moreover, within two years, 10 patients in the follow-up group progressed to CIN2 (progression rate, 15.15%), compared with only 1 patient in the conization group (progression rate, 0.26%) (*χ* ^2 =^ 51.68, *P*<0.01).

**Table 5:** Comparison of persistent/recurrent low-grade cervical intraepithelial neoplasia rates in patients receiving conization or follow-up.

Follow-up	Conization group (N=379)	Follow-up group (N=66)	*χ* ^2^	*P*
6 months	21.24%	78.79%	83.39	<0.01
12 months	15.97%	57.14%	47.34	<0.01
24 months	6.67%	23.21%	13.27	<0.01

## 4 Discussion

### 4.1 Management and potential risks of CIN1

The management of pathologically confirmed CIN1 is controversial, and the optimal treatment strategy remains unclear, although its management is becoming more conservative. The 2012 ASCCP guidelines (4), recommend observation rather than treatment, with a medical history review before biopsy and a hierarchical management approach. Patients with slight abnormalities, indicated by ASC-US or LSIL cytology, HPV16/18+, or persistent HPV infection, should also receive follow-up rather than treatment. Follow-up involves HPV testing combined with cytological screening after 12 months, with treatment (or further follow-up) only considered if CIN1 persists for at least two years. If cytological results are ASC-H or HSIL, as long as the colposcopy is satisfactory and endocervical curettage results are negative, there are three potential management approaches: combined screening at 12 and 24 months, diagnostic conization, or retrospective analysis of the cytological, histological, and colposcopy results to revise the diagnosis according to the ASCCP guidelines.

These recommendations assume the wide availability of sophisticated screening programs and the facilities and experts required to execute them, and may therefore be more applicable to developed than developing countries. Compared to developed countries, China has a relatively short history of routine screening. The number of qualified professionals and their expertise in cell pathology and colposcopy, clinical management capabilities, follow-up compliance, follow-up costs, and the psychological effects of follow-up are all factors that must be considered when deciding between treatment and follow-up, as they may affect the risk of missed diagnoses of high-level CIN in patients managed by follow-up only.

As early as 2010, Cheng et al. (11) reported that 31.0% of 274 patients with CIN1 diagnosed by colposcopy-directed biopsy and undergoing immediate conization were pathologically upgraded. Qian et al. (12) reported that after immediate LEEP, 52 of 109 CIN1 cases were upgraded to CIN2+, an upgrading rate of 47.71%. Chen et al. (13) reported that after immediate conization, 109 of 946 cases of CIN1 were upgraded to CIN2, an upgrading rate of 11.52%. Hu (14) reported that among 380 patients with CIN1 who underwent LEEP, 118 were upgraded to HSIL, an upgrading rate of 31.1%. In the present study, 466 patients with CIN1 underwent conization, 98 of whom were upgraded to CIN2+, an upgrading rate of 21.03%. Therefore, the reported pathological upgrading rate of CIN1 after immediate conization in China is high and variable, ranging from 10% to 50%. The observed variability may reflect a lack of cytological screening and colposcopy capabilities in China, with provision differing by region. In China, if patients with CIN1 are managed in accordance with ASCCP guidelines, most will receive only follow-up, bringing a significant risk of missed diagnosis of high-level CIN and increasing the risk of progression. When deciding treatment strategies, this information should be fully understood and considered, and personalized treatment should be administered based on the capabilities of the medical institution.

### 4.2 Factors related to pathological upgrading after conization for CIN1

Various factors have previously been associated with pathological upgrading. Cheng et al. (11) identified severe cytological abnormalities (HSIL or atypical glandular cells not otherwise specified) before colposcopy and dissatisfaction with colposcopy as independent factors influencing pathological upgrading after conization. Qian et al. (12) identified ASC-H/HSIL cytology, type III TZ, age (>40 years), and HR-HPV DNA load (≥100 pg/mL) as independent high-risk factors for pathological upgrading after conization. Chen et al. (13) considered ASC-US/HSIL cytology, unsatisfactory vaginal examination, menopause, and cervical endometrial biopsy independent high-risk factors for pathological upgrade after conization. Hu (14) found that ASC-H/HSIL/atypical glandular cells, HPV16/18+, and endocervical curettage pathological positivity were significantly correlated with postoperative pathological upgrading. Despite some inconsistencies, all studies identified an association between abnormal cytology and pathological upgrading after conization. However, there was no consensus on the minimum threshold for cytological abnormalities.

Analysis of preoperative cytology results in this study revealed significantly higher postoperative pathological upgrading rates in the ASC-H and HSIL groups than in the NILM group. Based on the results of this study and previous reports (12–14), we consider preoperative cytological ASC-H and HSIL findings the main factors influencing pathological upgrading after conization. In addition, we found that none of the patients preoperatively negative for HR-HPV showed pathological upgrading. Wang et al. (15) found that in patients with CIN1, the positive predictive value of preoperative HPV results for predicting CIN2+ was 16.4%, the negative predictive value was 94.1%, the specificity was 25.8%, and the sensitivity was 90.0%. They recommended that for patients with CIN1 confirmed by histopathology, follow-up should be considered if the HPV result is negative or only low-risk HPV infection is present, while for patients with HR-HPV infections (especially HPV16, 18, 51, 52, or 58), surgery or close follow-up can be chosen. As both HPV and cytology results influence pathological upgrading after conization, we conducted a stratified analysis of patients who underwent preoperative HPV and cytology screening, and found high pathological upgrading rates in the HPV (+)/ASC-H and HPV (+)/HSIL groups. This classification may help determine the risk of upgrading in patients with CIN1.

We also found that patients preoperatively positive for HPV but with normal cytological results had a relatively high upgrading rate of 11.86% after conization. A meta-analysis performed by Wang et al. (16) showed that in China, 20.39% of 5880 patients positive for HR-HPV but with normal cytological results had CIN2+ lesions identified by colposcopy-directed biopsy. In the United States, a study of 33858 women conducted by Stoler et al. (17) showed that the detection rates of CIN2+ and CIN3+ lesions in biopsies from patients positive for HR-HPV but with normal cytological results were 5.1% and 3.0%, respectively. A comparison of these two studies confirmed the insufficient screening capacity and number of cell pathologists in China (18), which increases the risk of upgrading for patients diagnosed with normal cytology, whether they are undergoing colposcopy-directed biopsy or conization. Therefore, the ASCCP guidelines cannot be fully adopted in China.

In recent years, HPV E6/E7 mRNA detection has become a research hotspot. Bruno MT et al. (19) found that HPV E6/E7 mRNA can be used in the triage of women with a borderline smear result, women positive in the HPV E6/E7 mRNA test had a greater risk of malignant progression of cervical lesions. Bruno et al. (20) also found that compared to HPV genotype determination, HPV E6/E7 mRNA detection had higher value in predicting higher-grade lesions. These suggested the clinical value of HPV E6/E7 mRNA detection in initial screening of cervical cancer. Further research is needed to determine whether HPV E6/E7 mRNA detection can be used for triage of pathologically confirmed CIN1.

### 4.3 Persistent/recurrent CIN and related factors in patients with CIN1 after conization

Our results showed that a proportion of patients were at risk of persistent/recurrent CIN after CIN1 conization. However, the persistent/recurrent CIN1 rate in the conization group was lower than that in the follow-up group at 6, 12, and 24 months. In addition, the risk of progression to CIN2 within two years was also lower in the conization group than in the follow-up group. Follow-up studies of patients with CIN1 carried out abroad also reported a CIN2+ incidence rate of close to 12% during a follow-up period of up to three years (21, 22). Therefore, surgical treatment of patients with CIN1 not only detects high-grade lesions that are missed in primary screening or colposcopy-directed biopsy but may also reduce the risk of progression to high-grade lesions in the medium-term.

Elit et al. (1) also compared the effects of regular colposcopy follow-up and immediate treatment in a randomized clinical trial involving 415 patients with CIN1 from Canada and Brazil. Of the 179 patients who underwent LEEP, 32 received pathological upgrading after surgery. During the 18 month follow-up period, three patients (1.7%) in the immediate treatment group and nine patients (4.4%) in the regular colposcopy follow-up group experienced disease progression. The risk of developing CIN2+ within 18 months was similar in both groups. Therefore, the authors considered an 18 month follow-up to be a reasonable management strategy for patients with CIN1 detected during cervical biopsy. However, we must consider the 32 cases of pathological upgrading, making a total of 35 cases of CIN2+ and a progression rate of 19.55% in the LEEP group.

We identified only preoperative HPV status as a possible influencing factor for persistent/recurrent CIN1 after conization. Söderlund-Strand et al. (23) followed 178 patients undergoing LEEP and identified nine cases of histologically confirmed persistent/recurrent CIN lesions. These nine patients had the same type of HR-HPV infection preoperatively and postoperatively. Among the 49 patients negative for preoperative HR-HPV, only one was found to have cytological abnormalities during postoperative follow-up, but the results of the colposcopy-directed biopsy were normal. Spinillo et al. (22) followed up patients receiving LEEP for persistent CIN1 and reported a postoperative persistent/recurrent CIN1 rate of 27.8%. The occurrence of persistent or multiple HR-HPV infections during follow-up was associated with postoperative persistent/recurrent CIN lesions. Therefore, we believe that patients with CIN1 and preoperative HR-HPV infection have a relatively higher risk of persistent/recurrent CIN1 after conization; this risk is further increased by persistent HR-HPV infection after conization. Owing to the limited sample sizes of these studies, further multicenter large-cohort trials are needed to verify the predictive value of HPV typing and quantification.

More than one-fifth of patients with CIN1 showed pathological upgrading after conization, and patients who received follow-up rather than surgery had a progression rate of over 10% within two years. Therefore, there is a certain risk of using follow-up management methods for CIN1 cases in developing countries or regions. Doctors should refer to the guidelines but comprehensively consider age, fertility requirements, preoperative HR-HPV and cytological results (especially high-level cytology reports), follow-up conditions, and other factors to determine the most appropriate treatment methods for patients with CIN1.

## Data Availability

The raw data supporting the conclusions of this article will be made available by the authors, without undue reservation.

## References

[1] Elit L Levine MN Julian JA Sellors JW Lytwyn A Chong S. Expectant management versus immediate treatment for low-grade cervical intraepithelial neoplasia: a randomized trial in Canada and Brazil. Cancer. (2011) 117:1438–45. doi: 10.1002/cncr.25635 21425144

[2] Wright TC Cox JT Massad LS Carlson J Twiggs LB Wilkinson EJ. 2001 consensus guidelines for the management of with cervical intraepithelial neoplasia. Am J Obstet Gynecol. (2003) 189:295–304. doi: 10.1067/mob.2003.633 12861176

[3] Wright TC Massad LS Dunton CJ Spitzer M Wilkinson EJ Solomon D. 2006 consensus guidelines for the management of women with cervical intraepithelial neoplasia or adenocarcinoma*in situ* . Am J Obstet Gynecol. (2007) 197:340–5. doi: 10.1016/j.ajog.2007.07.050 17904956

[4] Massad LS Einstein MH Huh WK Katki HA Kinney WK Schiffman M. 2012 updated consensus guidelines for the management of abnormal cervical cancer screening tests and cancer precursors. J Low Genit Tract Dis. (2013) 17:1–27. doi: 10.1097/LGT.0b013e318287d329 23519301

[5] Perkins RB Guido RS Castle PE. 2019 ASCCP risk-based management consensus guidelines committee. 2019 ASCCP risk-based management consensus guidelines for abnormal cervical cancer screening tests and cancer precursors. J Low Genit Tract Dis. (2020) 24:102–31. doi: 10.1097/LGT.0000000000000525 PMC7147428 32243307

[6] Solomon D Davey D Kurman R Moriarty A O’Connor D Prey M. The 2001 Bethesda System: terminology for reporting results of cervical cytology. JAMA. (2002) 287:2114–9. doi: 10.1001/jama.287.16.2114 11966386

[7] Nayar R Wilbur DC. The bethesda system for reporting cervical cytology: A historical perspective. Acta Cytol. (2017) 61:359–72. doi: 10.1159/000477556 28693017

[8] Pecorelli S Zigliani L Odicino F. Revised FIGO staging for carcinoma of the cervix. Int J Gynaecol Obstet. (2009) 105:107–8. doi: 10.1016/j.ijgo.2009.02.009 19342051

[9] Matsuo K Machida H Mandelbaum RS Konishi I Mikami d M. Validation of the 2018 FIGO cervical cancer staging system. Gynecol Oncol. (2019) 152:87–93. doi: 10.1016/j.ygyno.2018.10.026 30389105 PMC7528458

[10] Rodriguez-Manfredi A Alonso I del Pino M Fusté P Torné A Ordi J. Predictors of absence of cervical intraepithelial neoplasia in the conization specimen. Gynecol Oncol. (2013) 128:271–6. doi: 10.1016/j.ygyno.2012.10.020 23116936

[11] Cheng YF Wang XY Lv WG Cheng XD Xie X. Cervical intraepithelial neoplasia 2+ in low-grade squamous intraepithelial lesion pathologically diagnosed by colposcopy-assisted biopsy. Natl Med J China. (2010) 27:1882–5. doi: 10.3760/cma.j.issn.0376-2491.2010.27.003 20979903

[12] Qian M You ZX Yao FF Peng JJ Yan X Zhou BB. Analysis of missed CIN2+ in CIN1diagnosed by colposcopically directed biopsy. Chin J Clin Obstet Gynecol. (2013) 14:403–5. doi: 10.3969/j.issn.1672-1861.2013.05.006

[13] Chen Y Jiang W Zeng SY Liang MR Tu NT Zhu HT. Analysis of colposcopy biopsy pathology in the diagnosis of cervical low-grade squamous intraepithelial lesions(CIN1). Pract J Cancer. (2016) 31:1818–21. doi: 10.3969/j.issn.1001-5930.2016.11.023

[14] Hu R. Factor analysis of postoperative pathological progression in patients with low-grade cervical lessions. Zhengzhou: The first clinical college of Zhengzhou University (2019).

[15] Wang H He Z Han X Zhang D Zhang S. Prediction value with a novel and accurate tissue-based human papillomavirus detection method in low-grade squamous intraepithelial lesions. Cancer Med. (2022) 13:2576–87. doi: 10.1002/cam4.4634 PMC9249975 35343653

[16] Wang Z Liu T Wang YJ Gu Y Yang XH. Risk of cervical lesions in high-risk HPV positive women with normal cytology: a retrospective single-center study in China. Infect Agents Cancer. (2020) 34:1–9. doi: 10.1186/s13027-020-00291-x PMC7240930 32477424

[17] Stoler MH Wright TC Parvu V Yanson K Eckert K Kodsi S. HPV testing with 16, 18, and 45 genotyping stratifies cancer risk for women with normal cytology. Am J Clin Pathol. (2019) 4:433–42. doi: 10.1093/ajcp/aqy169 PMC6396747 30649177

[18] Research Report of the Pathology Artificial Intelligence Industry in China (2023). Available online at: https://www.cn-healthcare.com/articlewm/20200804/content-1135327.html. (accessed September 3rd, 2024).

[19] Bruno MT Ferrara M Fava V Barrasso G Panella MM. A prospective study of women with ASCUS or LSIL pap smears at baseline and HPV E6/E7 mRNA positive: a 3-year follow-up. Epidemiol Infect. (2018) 146:612–8. doi: 10.1017/S0950268818000250 PMC9134523 29465024

[20] Bruno MT Ferrara M Fava V Rapisarda A Coco A. HPV genotype determination and E6/E7 mRNA detection for management of HPV positive women. Virol J. (2018) 15:52. doi: 10.1186/s12985-018-0957-z 29587778 PMC5870089

[21] Cox JT Schiffman M Solomon D ASCUS-LSIL Triage Study (ALTS) Group. Prospective follow-up suggests similar risk of subsequent cervical intraepithelial neoplasia grade 2 or 3 among women with cervical intraepithelial neoplasia grade 1 or negative colposcopy and directed biopsy. Am J Obstet Gynecol. (2003) 188:1406–12. doi: 10.1067/mob.2003.461 12824970

[22] Spinillo A Gardella B Iacobone AD Stefania MD. Outcome of persistent low-Grade cervical intraepithelial neoplasia treated with loop electrosurgical excision procedure. J Low Genit Tract Dis. (2016) 20:307–11. doi: 10.1097/LGT.0000000000000242 27467826

[23] Söderlund-Strand A Kjellberg L Dillner J. Human papillomavirus type-specific persistence and recurrence after treatment for cervical dysplasia. J Med Virol. (2014) 86:634–41. doi: 10.1002/jmv.23806 24123176

